# Post-race reactions: The emotional paradox of high performance and anxiety – a conventional content analysis

**DOI:** 10.1186/s13102-024-00968-5

**Published:** 2024-08-28

**Authors:** Sofia Ryman Augustsson, Malin Bergh, Kornelia Petersson

**Affiliations:** Department of Sport Science, Faculty of Social Sciences, Linnaeus University, Kalmar, SE-391 82 Sweden

**Keywords:** Post-race blues, Endurance sports, Emotional well-being, Motivation, Performance, Goal-setting

## Abstract

**Background:**

Studies examining post-race emotional experiences in the context of endurance races among recreational athletes are scarce. The purpose of this study was to describe how recreational athletes experience the time after completing an endurance race.

**Methods:**

In this study, a qualitative study design was used, and data collection was carried out with semi-structured interviews. The selection of subjects was completed systematically through criterion selection. The selection criteria were men and women, aged 18 years or older, who had completed an endurance race, and finished, lasting at least 180 min within the past 12 months. The interviews were analyzed using conventional qualitative content analysis.

**Results:**

Sixteen recreational endurance athletes who, within six months, had completed an endurance race of running, cycling, cross-country skiing or Ironman, participated and were interviewed. Four overarching themes emerged from the analysis: “High on life”, “Loss of energy, Ambivalence and Melancholy”, “Activity-charged emotions”, and “Dimensions of emotions over time and new goals”, describing the content of the interviews.

**Conclusions:**

Endurance athletes experienced varied post-race emotions that were both physically and mentally challenging, suggesting a holistic approach to managing post-race emotions would be beneficial. From the athletes’ perspectives, post-race feelings were dependent on many factors, including time spent training for a specific race, and perceived inability to set new goals for an upcoming training period. Setting future goals prior to an event may be a tool for reducing the risk of negative post-race emotions, including post-race blues.

**Supplementary Information:**

The online version contains supplementary material available at 10.1186/s13102-024-00968-5.

## Background

There is an increased interest regarding the research area of mental well-being and illness for athletes [1–4]. For example, low subjective well-being has been proposed as a risk-factor for injury in sports [5] and a high degree of perceived external stressors are reported among youth elite athletes both in the sport context but also life events [4]. Well-being encompasses various aspects of individuals’ lives, including their physical health, mental health, and overall life satisfaction [6]. One theoretical framework related to well-being is the biopsychosocial model [7] which considers the interplay between biological, psychological, and social factors in understanding and explaining health and well-being. The model has been used and discussed in relation to sports rehabilitation and return to sports criteria [8, 9] and is valued for its holistic approach to optimal athletic performance beyond focus on just physical capabilities [10]. This model promotes a more comprehensive understanding of the athlete as a whole person, beyond just physical capabilities.

In endurance sports, studies have demonstrated how long races affect athletes’ physiological [11–13] and psychological [14, 15] profiles, and that social influences have a powerful impact on the athlete’s race experience [16]. For instance, elevated biomarkers for heart, liver, and kidneys after completing an endurance race in the form of an Ironman triathlon (3.8 km swim, 180 km bike, and 42 km run), were identified [11]. In another study [17], an increase in heart rate variability after ultramarathon races was observed, which was thought to be related to the significant physical strain. In addition, symptoms of depression and mood swings following competitions have been shown to be associated with the fatigue and sleep disturbances endured by endurance athletes during long ultramarathons [15]. Thus, the post-race period appears to be a vulnerable time for athletes in terms of mental health.

The phenomenon of ‘post-Olympic blues’ has previously been described, whereby athletes experience negative emotions and mental health issues following the Olympic Games [18, 19], and is characterized by a period marked by increased anxiety, depression, burnout, and challenges in adjusting back to everyday life. The period has been marked by anxiety, depression, burnout, and challenges in adjusting back to everyday life [19]. In addition, the degree to which athletes meet their performance expectations significantly affects their post-Games experience, with unmet expectations leading to various negative reactions. Still, the nature of ‘post-Olympic blues’ remains unclear and it has been suggested that future research is required the develop a clear definition of ‘post-Olympics blues’ [18]. Furthermore, ‘post-Olympic blues’ may be specific to elite athletes. As such, the post-game emotions of recreational athletes require investigation.

Another phenomenon described in the literature related to endurance sport, but not directly to elite athletes and success, is ‘post-race blues’. The term ‘post-race blues’ was defined by Galloway and Paul [20] as a psychological condition that can be equated with mild depression and feelings of emptiness after completing some form of endurance race. Roche [21] further describes that anxiety-like symptoms are one of the criteria for defining this issue. He also explains that there is no clinical definition of the phenomenon yet, but it is seen as a combination of various psychological principles. However, Galloway and Paul [20] argue that post-race blues definitely exist, and this reasoning is supported by Florio and Shapiro [22], who explains how athletes, regardless of their success, can experience depression, with feelings of loneliness and inadequacy after significant sporting achievements. In an interview with Emil Persson, the winner of the ski-race “Vasaloppet” 2023, describes his feelings afterwards. He names the symptom Vasallop’s blues and says “There will be an emptiness - What am I supposed to do now?” [23]. Other significant events, such as wedding and graduating, which typically bring something positive for the person in question, can be followed by a wave of negative emotions known as post-symptoms [24, 25]. These phenomena reinforce the theory that people can experience crisis-like states with associated depressive symptoms even after positive events.

Several studies have examined post-race moods [14, 26, 27], that could be associated with ‘post-race blues’, however the findings are equivocal. For examples, a study on ultramarathon runners found that post-race mood changes were more strongly associated with the difference between the athletes’ predicted and actual performance times rather than their perceived exertion levels [26]. The research also revealed that post-race mood disturbance, characterized by decreased energy, increased confusion, and elevated fatigue, was influenced by the accuracy of performance predictions [26]. Contrasting, another study found no changes in self-reported depression scores in relation to ultra-endurance race outcomes [27]. In this pilot study, 33 ultra-distance triathletes were assessed with the Beck Depression Index (BDI-II) before and after an ultra-distance triathlon. The majority of participants showed no symptoms of depression, either before or after the race.

Importantly, most previous studies on post-race moods and ‘blues’ have used quantitative study designs. To the best of our knowledge, qualitative studies examining post-race experiences in the context of endurance race among recreational athletes are scarce and studies that have described emotional aspects related to endurance sports appear limited to qualitative studies with a ‘bottom-up approach’, where coding categories are derived directly and inductively from the raw data. The purpose of the study was to explore and describe how recreational athletes experience the period after completing an endurance race and capture their thoughts about post-race blues using a conventional content analysis design.

## Methods

### Study design and setting

In order to best answer the study’s purpose; to explore and describe how recreational athletes experience the period after completing an endurance race, a qualitative interview study design with an inductive approach was used. Qualitative methods allow for the illumination of the individual’s perspectives, experiences, and emotions, which are crucial in this study [28]. The authors intended to explore and describe individuals’ post-race experiences with the goal of providing a broader understanding of post-race emotions and reactions. This study aimed to describe how recreational athletes experience both emotionally and physically feelings after endurance races lasting over three hours race and their thoughts about post-race blues. Post-race blues, in this study, are defined as feelings of emptiness, negatively affected emotional well-being comparable to mild-to-moderate depressive symptoms, and/or feelings of meaninglessness after completing an endurance race [20]. The study was carried out at the Department of Sports Science, Linnaeus University and the interviews were conducted by video communications during January to March 2023.

### Participants and recruitment process

The initial recruitment attempt was conducted through social media platforms such as Facebook and Instagram. In addition, an information letter was emailed to two local sports clubs requesting that they share the study information with their members. The selection of subjects was completed systematically through criterion selection, in order to include those who were suitable to participate [28]. The inclusion criteria were: (1) men and women, (2) aged 18 years or older, (3) who had completed and finished an endurance race lasting at least 180 min within the past 12 months. As the study focused on the experiences of recreational athletes, individuals who were engaged in elite training or competed at an elite level were excluded. Athletes whose race had been negatively affected by illness or injury were also excluded. A total of 16 athletes were recruited and none of these were excluded. All athletes achieved written information and informed consent was obtained. The study was approved by the Swedish Ethical Review Authority (DNR 2023-02058-02) and the investigation conforms to the principles outlined in the Declaration of Helsinki [29].

### The interview guide and pilot testing

The authors initially developed a semi-structured interview guide. The interview guide consisted of questions addressing ordinary experience of training/sports, post-race experience and emotions, well-being, motivation and thoughts about post-race blues (Additional file 1). To ensure that the interview guide responded to the purpose of the study and to minimize misunderstandings and misinterpretations of the questions a pilot interview was conducted. After this interview, some minor changes were made to the interview guide. The pilot interview was not used in the data analysis.

### Procedure

Data was collected using semi-structured interviews with the aid of the interview guide. The interviews were conducted in the athletes’ native language and the text was then translated. All interviews were conducted digitally using the platforms Zoom Video Communications, FaceTime, or video chat on Messenger and lasted for approximately 20 min and were recorded using the voice memo application. In order to address questions about emotional experiences in the weeks following the endurance race, the interviews were conducted at least three weeks after the completion of the race. After each interview, verbatim transcriptions were made by the authors (M.B. & K.P.). The interviews were then re-listened to for the purpose of correcting any errors or shortages, thus enhancing the quality and value of the data.

### Data analysis

The data processing followed the steps of a qualitative conventional content analysis [30]. We chose to use a conventional content analysis as the goal was to describe emotions based on the participants’ own voices and to provide a description of the reality as they are lived out in a particular setting. The analysis process was inductive, and the authors did not derive categories from existing theories, nor did they intend to verify existing theories. Thus, the authors did not have any specific expectations for the data before the analysis started. Rather, we expected that themes related to post-race experience of endurance race would emerge from the texts. Once all the interviews were transcribed, they were read multiple times by the authors to gain an overall impression of the material and to identify similarities and differences [31]. The authors approached the text by making notes of their first impressions, thoughts, and initial analysis. This was followed by the identification of meaning units addressing the aim of the study. The authors also selected units with recurring essence among the athletes, as well as experiences that differed [31]. The units were condensed and labelled with a code. Next, the various codes were compared on the basis of differences and similarities and sorted into eleven categories describing the manifest content. The categories were expected to be exclusive (distinct from each other) and exhaustive. Finally overarching themes were developed to describe the latent content, i.e., the underlying meaning in the manifest content [30]. The interviews were re-read and re-listened several times for reflection and to ensure that the themes matched the purpose of the study and that the meaning units could be outlined in the themes. All authors took part in the analysis and continually discussed, in an open and critical dialog, all the steps in the analysis process, checking the alignment of codes, categories and quotes, and cross-checked for agreement, until consensus was attained [32]. Examples of process of the analysis are shown in Table 1.

**Table 1:** Examples of the analysis process from meaning unit to code

Meaning unit	Condensed meaning unit	Code
I would probably describe that as quite empty, as a void I would say. It will be a bit this feeling, well what do I do now?	It felt empty	Emptiness
It took several weeks…0.3–4 weeks, before I could recognize myself…	3–4 weeks, before I could recognize myself	Time

## Results

### Participants

In total, 16 recreational athletes, comprising 10 men and 6 women, aged 23 to 59 years (mean age 40.5 (± 10) years), agreed to participate in the study. All of these met the eligibility criteria and were included in the study. The athletes trained approximately between 2 and 12 h per week during the last 3 months prior to the race and competed in running, cycling, cross-country skiing or Ironman (Table 2) with a duration of the races ranging from 4 to 27.5 h within the last 6 months.

**Table 2:** Sex, age range and type of race and distance for those on which the interviews are based

Participant (No)	Sex	Age range	Type of race	Total distance (km)
1	Man	20–30	Ski race - Vasaloppet	90
2	Man	31–40	Ironman	226
3	Woman	20–30	Ski-race - Halvvasan	45
4	Woman	51–60	Ski-race -Tjejvasan	30
5	Man	31–40	Ski race –Kortvasan	30
6	Man	31–40	Ironman	226
7	Man	51–60	Running race	50
8	Woman	31–40	Ski race - Vasaloppet	90
9	Woman	20–30	Ski race - Vasaloppet	90
10	Woman	41–50	Ironman	226
11	Man	51–60	Ski race - Vasaloppet	90
12	Man	51–60	Bike race - Vätternrundan	315
13	Man	51–60	Ironman	226
14	Man	20–30	Bike race - Vätternrundan	315
15	Man	41–50	Ironman	226
16	Woman	31–40	Ironman	226

### Analysis

A total of four overarching themes emerged: “High on life”, “Loss of energy, Ambivalence and Melancholy”, “Activity-charged emotions”, “Dimensions of emotions over time and New goals”, which describe the content of the interviews (Table 3).

**Table 3:** Examples of condensed meaning units, codes, categories and themes from the present data

Condensed mening unit	Code	Category	Theme
But now I’m happy with it.	Satisfied	Performance satisfaction	High on life
I felt like I could handle anything.	Handles everything	Invincible	
You’re so drained, and it’s clear you’re running on low energy.	Low energy	General exhaustion	
I have had a hard time continuing	Unmotivated	Reduced motivation	Loss of energy, Ambivalence and Melancholy
You start to question yourself	Ambivalent	Ambivalent feelings	
It felt empty	Void	Sense of emptiness	
There can be some anxious feelings when I’m out running	Anxiety	Emotionally charged	
To perform, you build up some kind of pressure on yourself	Performance	Performance focus	Activity-charged emotions
You plan your everyday life to catch up with the training	Planning and adaptation	Adjustments in life	
3–4 weeks, before I could recognize myself	Time	Different Time Aspects	Dimensions of emotions over time and New goals
The motivation for training has increased, I want to train for something new	New motivation	New objective	

### High on life

This theme has been formulated based on categories that describe a sense of pride and positive emotions after the race. “High on life” was emerged from two categories: *Performance satisfaction* and *Invincible*.

### Performance satisfaction

All athletes in the study expressed satisfaction with their completed race, regardless of the time taken.

“It’s really tough during the race, but I had a positive feeling, and most importantly, I never felt like giving up or quitting. I felt like I could get through this. And I carried that feeling with me afterwards.” (No. 10).

“But in the days after the race, I was really happy. And I’m so excited for the next goal.” (No. 5).

Some athletes simply answered the interview question about whether they were satisfied with their race without providing a deeper explanation.

“Yes, I am.” (No. 11).

“Yes, oh God, I was.” (No. 15).

### Invincible

Athletes experienced euphoria and a feeling of invincibility after completing the race, which in several cases lasted for a period of time after the race.

“So, right after, there’s a small feeling of being euphoric, a feeling of achieving something. And when you go pretty fast and you tell someone who knows about the Vätternrundan, they go, ‘Wow, that was fast,’ and then you put your own achievement into perspective… you might even get a bit cocky right there.” (No. 14).

“Well, you kind of get high on life, or I got high on life. I felt invincible and that I can overcome anything that comes my way.” (No. 2).

### Loss of energy, ambivalence, and melancholy

This theme is shaped by categories that describe the athletes’ physical and mental experiences after completing a race. Most of these experiences were expressed in athletes representing longer race, e.g. Ironman. These are interrelated due to the situation and feelings of being an endurance athlete and consist of the following categories: *General exhaustion*,* Reduced motivation*,* Ambivalent feelings*,* and Sense of emptiness.*

### General exhaustion

Several athletes described both physical and mental exhaustion symptoms in the period after completing an endurance race. They also expressed that the physical exhaustion made it difficult to distinguish from their mental well-being.

“I think it’s a combination that, in the beginning, you get an endorphin kick, and then you do a long race that exhausts your body and mind, which makes it easier to enter a phase where you feel like, ‘I’ve done this and can handle this physically, but my body is so drained that I can’t handle it mentally.‘” (No. 9).

Some athletes attempted to resume training earlier than what their bodies allowed, which may also indicate that physical and mental well-being do not always align.

“Yes, the day after, I went out to try jogging, which I soon realized was foolish because my body was completely depleted. So, I had the urge to train already the day after, but then I realized I need to be smarter.” (No. 13).

“I wanted to get up and train right away, but I couldn’t. Because I had so much pain in my knees, hips, well, even shoulder blades, sternum, and neck, basically everything hurts a lot, so I just thought, no, I need to rest… It took me a couple of weeks after the race to overcome that feeling. When I had recovered physically and could go for my first run without pain… which is a bit sad because running is what I really love, but… I haven’t been doing much cycling and such during the winter, and I probably won’t cycle again.” (No. 16).

### Reduced motivation

Several athletes described a nonexistent or reduced motivation to engage in training after the race. This applied to both discipline-specific training and general exercise. For some athletes, the reduced motivation persisted for a longer period afterward. Several of them mentioned that despite the lack of motivation, they still carried out their training out of routine or discipline.

“Well, I would say that my motivation to train still feels very low. I do train once a week, but it’s not because I necessarily want to. It’s more to stay active and maintain what I have built up so far. I’m trying to focus on the next race.” (No. 8).

“Yeah, I felt that. Actually, for a couple of weeks afterward. But I know that I feel good when I exercise, so I started my running training a couple of weeks after the race. I had enough of cycling, and swimming wasn’t an option either. But running, well, I’m going to run the Stockholm Marathon, so maybe around 14 days or three weeks later, I was fed up with training, and now I’m doing more casual workouts. I didn’t become a couch potato afterward, but my training was really at a low level. About three weeks later.” (No. 12).

“ I don’t feel any motivation to do it, really. It’s running that I had a bit of a preference for before, but I absolutely don’t feel like doing any more cycling. Swimming was fantastic, I had a great experience with it during the race.” (No. 16)”.

### Ambivalent feelings

The athletes described an emotional paradox where positive and negative emotions wrestled with each other. All the athletes were familiar with the phenomenon post-race blues and related some of their post-race feelings to the phenomenon. In the immediately after the race, most athletes felt positive and satisfied. However, over time, these feelings diminished, giving way to negative emotions, difficulties in relating to their own feelings, and/or a lack of motivation. The athletes expressed that it was challenging to know what would happen next and that they almost missed having some guidance in their training.

“I felt, at least, that I had checked something off….and like, well, now it’s done! But then, you start to feel a bit restless, questioning yourself, what are you doing and what should you do now. Because it’s such a long process of training for such a race. I mean, I signed up for this in 2020, even in 2019. There has been a long wait for this race and this competition, so many things need to settle in your mind. And then, especially after about four, five weeks, you start to think, okay, what should I do now? Because you feel a bit lost in terms of where you want to go with your training, at least.” (No. 2).

“But then, after a while, I could relate the feelings that arose to the feelings I have when working on a dance performance for a very long time, and then it’s all over in a weekend. But oh, right, I’ve been thinking about this for a year, and now I’m not going to do it anymore. But for me, it quickly turned into a craving for more races, and I could do this again.” (No. 9).

“No, right after, you say, like you’ve said every year, no, not again next year. Not again, it was tough and not fun. But then, it creeps closer, and you may be asked if you want to help again….we all help each other. So you discuss it in the group, and when they ask if you’re going to participate, you think, well, then I’ll join too. So even if you say never again, here I am, getting ready to do it again.” (No. 14).

### Sense of emptiness

Regardless of whether athletes experienced negative or positive emotions after the race, seven of them described a feeling of emptiness, void, or mild depressive symptoms. Mood swings, difficulties in recognizing themselves in everyday life, or feelings of sadness were reported by several athletes.

“But mentally, it’s often or always for me a void… like, okay, now what? It’s like this buildup tension that then becomes a relief and then it almost feels like mild depressive symptoms.” (No. 15).

Some attributed this emptiness to the intense release that the race represented, while others believed it could be correlated with the fact that they had been looking forward to something for a long time, and now it’s over without a concrete event to look forward to in the near future. The feeling of emptiness is one of the symptoms of post-race blues.

“For the first five days, I didn’t feel anything special except that I felt a bit empty. Then, I don’t know if it’s related to my mental state, but the week after, I was in a really bad mood. I don’t know why, but it was an unusually bad mood for me because it was like going from intense training to not training at all. I think it was quite evident in my mood and temperament. I was very irritable.” (No. 8).

“I would say that feeling of emptiness after completing a big race. I mean, if you’ve had a big goal for a long time that you’ve also dedicated your life to, and then once you’ve accomplished it, that goal is no longer there.” (No. 1).

### Activity-charged emotions

The theme describes how the athletes experienced intense emotions in various contexts related to the race or the time afterward. This resulted in three categories: *Emotionally charged*,* Performance focus*,* and Adjustments in life.*

### Emotionally charged

Athletes described experiencing intense emotions regarding continued training for a specific discipline. A couple of athletes mentioned the need to take a break from discipline-specific training for a period.

“After the long race, I didn’t feel like swimming anymore, but in general, I want to train, it’s just that swimming hasn’t come back yet. Fortunately, I’m excited about running and cycling.” (No. 15).

“I have to say that, well, I feel quite done with cross-country skiing after the race, actually. Of course, I would like to do it again next winter, but for now, I feel completely finished with it.” (No. 8).

One athlete reflected on the importance of being able to train for enjoyment rather than solely focusing on performance, while others described allowing motivation to guide their training for a period.

“However, I have gained a deeper understanding that when I initially started enjoying, in this case, cross-country skiing, it could be more about enjoying the training itself. I shouldn’t just train for the sake of training but rather keep myself active, more like that, and that feeling disappeared a bit when I signed up for a race. Now I can enjoy it a bit more, and maybe I have gained a greater insight into the importance of doing both, even before a race. So I’ll take that with me, that I will do it again, and I can enjoy it even during the process because it’s part of the preparation.” (No. 9).

### Performance focus

Several athletes described experiencing performance anxiety before, after, or reflecting on their performance afterward. Some mentioned that their performance was influenced by external factors beyond their control, leading to a certain level of frustration. Others felt that their performance aligned well with their preparations.

“Yeah, now afterward, I am, at that moment, I had some naive thought that I would finish it in 12 hours, but now I’m satisfied with it.” (No. 12).

“…I could definitely have wished to do better, but I was happy that I crossed the finish line. That was actually what I aimed for.” (No. 3).

“As a competitive person, I can’t be overly satisfied, but I am definitely content with the conditions I had for the day.” (No. 1).

### Adjustments in life

The interviews also revealed that the athletes’ lives must be adapted in various ways to find time for training, avoid getting sick, or negatively affecting their performance before the race. The athletes also mentioned that they had been looking forward to the races for a long time, discussing them, and planning for them, which had an impact on their daily lives for an extended period.

“Because you have that goal and you’re training for it and talking about it and all that. And then, when it’s finally over, you might actually feel a sense of relief, maybe that too, because you have accomplished it. And yes, with age and everything, you think it’s getting worse and worse, but it’s absolutely not. You’ve filled everything with this, and your thoughts have revolved around the race, so there’s a sense of emptiness, and maybe that’s why you sign up for the next thing, I don’t know.” (No. 7).

“But then I think that leading up to the race, it has been a topic of conversation, it has been my focus, I have chosen to prioritize and exclude or include everything in my daily life for it, like ‘I probably shouldn’t drink’ or ‘I probably shouldn’t do that.’ Or ‘I need to train’ or if I got sick, it was like I have to get well for this, and it has been such a big thing, and now it’s gone.” (No. 9).

“You think like this: ‘Well, now I’m going to focus on the Vasaloppet next year.’ You think about it the whole year, and then either way, regardless of how it goes, you just… I think it’s a bit similar at work, you know, if you have a task to complete. And then you do it. And if you don’t have anything going on, there’s a bit of emptiness, of course.” (No. 5).

### Dimensions of emotions over time and new goals

This theme encompasses the time aspect of the athletes’ emotional impact after the race, regardless of positive or negative emotions. The theme aims to highlight that some athletes experienced a significant change in their mental well-being for an extended period after the race. The theme also describes how the athletes discuss how their motivation is positively influenced by setting new goals or signing up for new races in the near future. This overarching theme consists of the categories: *Different Time Aspects* and *New objective.*

### Different time aspects

Athletes’ emotional well-being after racing varied greatly, as did the duration of the perceived impact on their well-being. Below are some examples where athletes reported their well-being being restored within the first couple of weeks after completing the race.

“About two weeks.” (No. 9).

“It usually sorts itself out, but I start looking for new things, so the first few weeks are down weeks. " (No. 15).

“It was probably a week.” (No. 8).

There were also athletes who experienced lingering emotional impact for a longer period after racing. The following quotes exemplify athletes who felt emotional effects for 4–8 weeks after completing the race.

“It’s still there, actually. When I think about it, I get a positive feeling, (laughter).” (No. 10).

“It took me a while to kind of let everything sink in and settle down. So it probably took those four weeks, if not more, for me to land in something that felt normal, so to speak.” (No. 2).

### New objective

Some athletes described that their motivation to participate in a similar race or a new different race had increased as a result of completing the previous race, but also that new goals were essential to manage negative emotions.

“The motivation is high! Absolutely! I would say that it’s almost even higher motivation to complete the entire Vasaloppet, to take the next step. Because it felt so good, you want to experience that feeling of crossing the finish line again.” (No. 8).

“Yeah, right after, I was actually motivated to train even more because I thought, ‘Wow, I’ve become really good at this, now I want to become even better.’ So, it wasn’t about taking a break; it was more like, ‘Great job!’ and then looking at the data and thinking about how much better I can become.” (No. 14).

“But then I start planning for the next race, almost to get out of the ongoing slump.” (No. 15).

“In my case, no, I actually just wanted to do it as quickly as possible, partly because I signed up again for the same race and had this new goal to strive for. So, I was quite motivated to start over, to embark on this journey once again. And then you get that glimmer of light in the future, you know.” (No. 1).

## Discussion

The analysis revealed that most athletes in the present study experienced emotional alterations after a race, some of which could be linked to post-race blues. The post-race feelings varied over time, from euphoria to anxiety, and between athletes and were experienced as both physically and mentally challenging. A total of four overarching themes emerged that addressed the purpose and research questions of the study. The themes were as follows: ‘*High on life’*,* ‘Loss of energy*,* Ambivalence and Melancholy*,* ambivalence and melancholy’*,* ‘Emotionally charged feelings’*,* and ‘Dimensions of emotions over time and new goals’*.

In the theme ‘*High on life*’, athletes stated feelings of strong well-being, euphoria and invincibility which may be related to the phenomenon of ‘runner’s high’. ‘Runner’s high’, is a powerful euphoria during long-distance running which has previously been noted to correlate to an increased release of endocannabinoid (eCB) hormones [33]. In the present study, several athletes described an experience of being “high” on endorphins, which could be attributed to the release of eCB. It is possible that the athletes who experienced strong positive emotions and enhanced emotional well-being, feeling “high on life” in the period after the race, had a significant release of eCB or other substances and hormones that can affect a psychological reaction. The pride of completing the race and achieving their goal can also be attributed as a contributing factor to the positive emotional well-being. Feeling of pride is a positive emotional reaction to a personal success which often are related to achieving goals in sports. Athletes’ motivation and goal orientation is vital for performance and athletic emotional well-being [34]. All athletes described that they felt satisfied with their race performance, which, in several cases, resulted in an enhanced positive experience. However, no concrete explanations were provided as to why some athletes experienced this strong positive emotional impact. Nevertheless, one inclusion criterion was for the athlete to have completed and finished an endurance race. An athlete who has made a huge effort to complete a race will be motivated to explain his or her effort positively.

In contrast to the findings mentioned above, experiences of a negative nature were also noted. Some athletes described negative effects on their emotional well-being after the race, which were presented in the theme of ‘*Loss of energy*,* ambivalence and melancholy’*. It is also important to note that age was not the focus of the study. Some athletes experienced significant physical exhaustion, mixed feelings about their participation, and a sense of emptiness after dedicating a significant part of their lives to the race, which was now over. Other found it difficult to determine whether the physical exhaustion affected their mental well-being or vice versa. These results are similar to what Anglem et al. [15] found in their study, where they observed that depressive symptoms can be related to the fatigue and exhaustion that endurance athletes experience during long races. Thus, it may be difficult to distinguish between general fatigue and emotional well-being.

The definition of post-race blues used in this study refers to a psychological state that can be likened to mild depression and feelings of emptiness [20]. The theme of energy loss, ambivalence, and melancholy presents a subtheme related to feelings of emptiness. Several participants described a sense of emptiness, a void, and a lack of guidance after completing their races. Most of the feelings related to emptiness were expressed in athletes participating in longer races, e.g. Ironman. This may be due to longer preparation and greater achievement. These feelings align with what Howells & Lucassen [19] found in their study on post-Olympic blues, where athletes reported feelings of emptiness and depressive symptoms after completing the Olympics. One could argue that there is no greater event than the Olympics, and the participants in the study by Howells & Lucassen mentioned that the time previously devoted to training was now freed up which was a contributing factor to the perceived sense of emptiness [19]. This phenomenon is also explained as the effort being almost a training addiction and a driving force that has characterized the athlete’s entire life situation for a long time [35, 36]. Future studies may be advised to investigate the influence of the duration on post-race emotions.

The category of reduced motivation consists of experiences of a lack of training motivation after the race. Some attribute this to being highly driven by motivation and not having a goal in mind, while others felt the need to take a break to recharge before embarking on something new. A few athletes described that training was no longer important to them since they had already completed their race, which was their main goal.

Another theme that emerged was ‘*Emotionally charged feelings*‘ experienced by a few athletes after the race. These symptoms were primarily identified within the specific sport discipline in which the endurance race was conducted. One athlete experienced significant physical exhaustion, resulting in the need for hospitalization for a period afterward. Despite feelings of anxiety, the athlete describes a sense of pride and satisfaction in having completed the race and also expresses euphoria and joy in this regard. It has previously been stated that mental toughness, resilience, passion, and a dependence on running are important psychological factors for successfully completing ultra-distance races [37]. Since all athletes in the present study managed to finish their races, they probably possess the required psychological qualities for the individual challenge. Still, in the theme ‘*Ambivalent feelings*‘, it was described that these positive feelings weakened, giving way to negative emotions and lack of motivation.

The overarching theme ‘*Activity-charged emotions*‘ also includes categories involving performance anxiety. Some athletes describe a desire to quickly beat old times, start once again, and perform at a high level yet again. Some athletes described their emotional well-being being significantly affected after the race to large extent. These feelings varied from mild to moderate, with some athletes experiencing an inferior emotional well-being for an extended period.

It was noted that the duration of athletes’ changed well-being, whether positive or negative, varied, which is described in the theme ‘*Dimensions of emotions over time and new goals*‘. Athletes who experienced a significant impact on their well-being were affected for at least one week up to eight weeks after the endurance race. Anglem et al. [15] noted that ultra-runners’ emotional well-being stabilized after 14 days. This result partially aligns with the findings of our study. However, several athletes experienced an impact for a longer period, ranging from 3–4 weeks up to 8 weeks in some cases. On the other hand, some described an unchanged emotional well-being in the period after their races and considered themselves unaffected, apart from a sense of satisfaction with their performance. The theme also describes how athletes experienced a significant increase in motivation to continue their training and participate in new races in the near future. Some also mentioned that participating in new races is a continuous strategy to maintain training motivation over time and registering for a new race is a way to manage the symptoms, reduce the feeling of emptiness, and recharge. Others were familiar with the phenomenon from previous experiences and were prepared for how it might feel afterward, thus having strategies to overcome the period of post-race blues. In many cases, these strategies involved registering for new races, setting new goals, or accepting that the feelings would dissipate over time. The phenomenon of new goal setting has previous been described in a study on Olympic snowboard athletes [38]. In this study, it was reported that there can be an “emptiness vacuum” unless the athlete sets a new goal [38]. Thus, setting new goal are most likely vital to cope with post-race emotions and regaining training motivation.

In the study, some athletes expressed a desire to take on bigger, longer, and new races, possibly due to their self-efficacy increasing significantly as a result of their previous race, leading to a strong motivation to take the next step. Completing a race can increase self-efficacy, and individuals may then be motivated to undertake even more challenging endeavors. A few athletes experienced no change in motivation after completing their endurance race. Some attributed this to addiction, while others explained it as having routines, such as attending group training sessions on certain days every week or training with acquaintances on fixed days and times.

In summary, the themes emerged from the present data acknowledges both physical and psychological feelings associated with the athletes’ competition, training preparation and performance. In general, athletes described high-pressure situations, and expressed an emotional paradox with both positive and negative feelings. The categories “New objective and “Performance focus” describe experiencing of both performance anxiety and the significance of new goals to manage negative emotions. It was also reflected on the importance of motivation and being able to train for pleasure for a period after a race. Secondly, in the category “adjustment in life” athletes described that they had to prioritize and exclude things in daily life underscoring the cultural context in which they compete. Managing social pressure seem to be of importance for these athletes but also the social support. The social context needs to be further investigated, especially for recreational athletes who spend a lot of time training in addition to perform regular work. Taken together, feelings that are associated to physical, mental and social conditions seem to be present in athletes as post-race experience. Thus, athletes may benefit from a plan for coping with these feelings and the adjustment in everyday situations.

### Methodological considerations

A qualitative conventional content analysis was chosen which involves extracting relevant information directly from the study participants. In a conventional content analysis, categories are derived from data during data analysis [30]. The information is solely based on the participants’ own experiences and perspectives and theoretically predetermined perspectives are not being used [30]. To assure internal validity from the present study, we used the common criteria for dependability, credibility and transferability [31]. Athletes were selected using inclusion and exclusion criteria in the recruitment process and selected purposefully, which provides the power to select information-rich cases [28]. These criteria were established to ensure that the athletes in the study met the necessary requirements to investigate the intended research topic. Thus, the athletes were required to have completed an endurance race lasting at least 180 min to ensure that experiences of a long-distance race were covered. To ensure recollection of feelings the race should have been performed within the past 12 months. It has previously been noted that mood changes seem to be evident rather quickly after racing, and usually resolved within 2 weeks [15]. Thus, the interviews were conducted at least three weeks after the completion of the race to make sure that most emotional experiences were expressed. However, as the literature is scarce when it comes to the phenomenon of post-race blues, we cannot be certain when these feelings have passed. In addition, the time frame for the race and the interviews differed between the athletes. Although the interviews were conducted no later than 6 months after the race, it cannot be guaranteed that all athletes remembered all emotions. Some recollections may have altered and being mixed up with new experience when preparing for another competition.

To ensure that the interview guide responded to the purpose of the study and to minimize misunderstandings and misinterpretations of the questions a pilot interview was conducted. The use of a semi-structured interview guide allows for follow-up questions when the researchers feel that the answers provided are insufficient or that further elaboration would add value to the study. To minimize the risks of bias, erroneous conclusions, or manipulated interviews, the authors reviewed each other’s conducted interviews and read each other’s transcriptions multiple times.

One limitation of the present study may be that there were significant individual differences in the duration of the races, ranging from 4 to 27.5 h, which naturally led to different experiences after the races. It is possible that the amount of time and focus dedicated to preparing for an endurance race may have influenced the experience of post-race experience. Thus, a more homogeneous group of participants would have been desirable. In addition, differences in type of races and sports (running, cycling, cross-country skiing, and triathlon) involved in the study may weaken the generalizability of the results. For example, the athletes who completed a cross-country skiing race discussed how their motivation for continued skiing each season is affected by the diminishing conditions as the weather gets warmer. Thus, discussing specific motivations for training in the same sport/sports as the race are challenging to generalize. The athletes’ private lives and environmental factors may also pose limitations in the study. The researchers cannot guarantee that the experiences associated with post-race emotions are not influenced by the athletes’ overall life situation. Furthermore, other factors such as gender may also have influenced the results. It has previously been noted that there are differences between gender when it comes to coping with performance stress in sports [39]. For example, it has been suggested that female athletes search for social support to manage goal frustration to a higher degree than their male counterparts [39]. However, due to the qualitative study design used in the present study no gender differences could be examined and specified. Still, in the current study, the themes that emerged were based on experiences and feelings described by both male and female athletes and no themes were based on statement from only one gender.

## Conclusions

The endurance athletes experienced varied post-race emotions that were both physically and mentally challenging, suggesting a holistic approach to managing post-race emotions would be beneficial. From the athletes’ perspectives, post-race feelings were dependent on many factors, including time spent training for a specific race, and perceived inability to set new goals for an upcoming training period. Setting future goals prior to an event may be a tool for reducing the risk of negative post-race emotions, including post-race blues. Our understanding of the post-race blues is in its beginnings and this study provides background for further research, specifically in terms of the recreational endurance athletes and emphasizes the need of prospective and intervention studies that focus on risk and protective factor for post-race blues.

### Electronic supplementary material

Below is the link to the electronic supplementary material.

Supplementary Material 1

## Data Availability

The data collected and analyzed in the current study are not publicly available due to ethical restrictions, but are available from the corresponding author upon reasonable request.
