# Supplementary material for: Post-race reactions: The emotional paradox of high performance and anxiety – a conventional content analysis
Source: BMC Sports Sci Med Rehabil. 2024 Aug 28;16:183. doi: 10.1186/s13102-024-00968-5 (PMC11360306; doi:10.1186/s13102-024-00968-5)
Supplement: Supplementary file 1 — Supplementary Material 1 [file 13102_2024_968_MOESM1_ESM.docx]

**Additional file 1.** The interview guide.

**Initial questions:**

1. **What are your experiences with training/sports?**

-Approximately how many endurance races have you completed in total?

-Type of race, duration and how long have you been involved in this form of sports?

1. **Which race are you referring to in our conversation? Can you describe this race?**

-How long, when was this carried out, time frame?

1. **Were you satisfied with your performance?**

-why? Describe.

1. **Approximately, how many hours a week did you train before the race (based on the last three months).**

-any challenges?

**Questions referring to the experience of competing in endurance race:**

1. **Can you describe your mentally and physically well-being from one week after the race to 2 weeks after the race?**

- feelings? - experience of mental condition?

- Experience of changes during these weeks? How? Positive/negative? Challenges?

-If the mental well-being was affected, how long did it take before you felt "normal" again? -Why do you think the race affected your mental well-being?

- Other things, apart from the race, that influenced?

1. **Have you heard of the phenomenon post-race blues? What are your thoughts about post-race blues?**

- If yes – can you describe? (Interviewer corrects any major misunderstanding)

- If no, the interviewer describes the phenomenon.

-Believes? Experience?

1. **Describe your motivation for general training after completing an endurance race?**

-Motivation for continuing to train in the same type of sports? Compete in the same kind of race?

1. **Any further information/experience?**
